# Supplementary material for: The causal relationship between the human gut microbiota and pyogenic arthritis: a Mendelian randomization study
Source: Front Cell Infect Microbiol. 2024 Nov 26;14:1452480. doi: 10.3389/fcimb.2024.1452480 (PMC11629706; doi:10.3389/fcimb.2024.1452480)
Supplement: Supplementary file 1 [file DataSheet1.zip › mendelian test/ebi-a-GCST90027826.csv_leave_one_out.pdf]

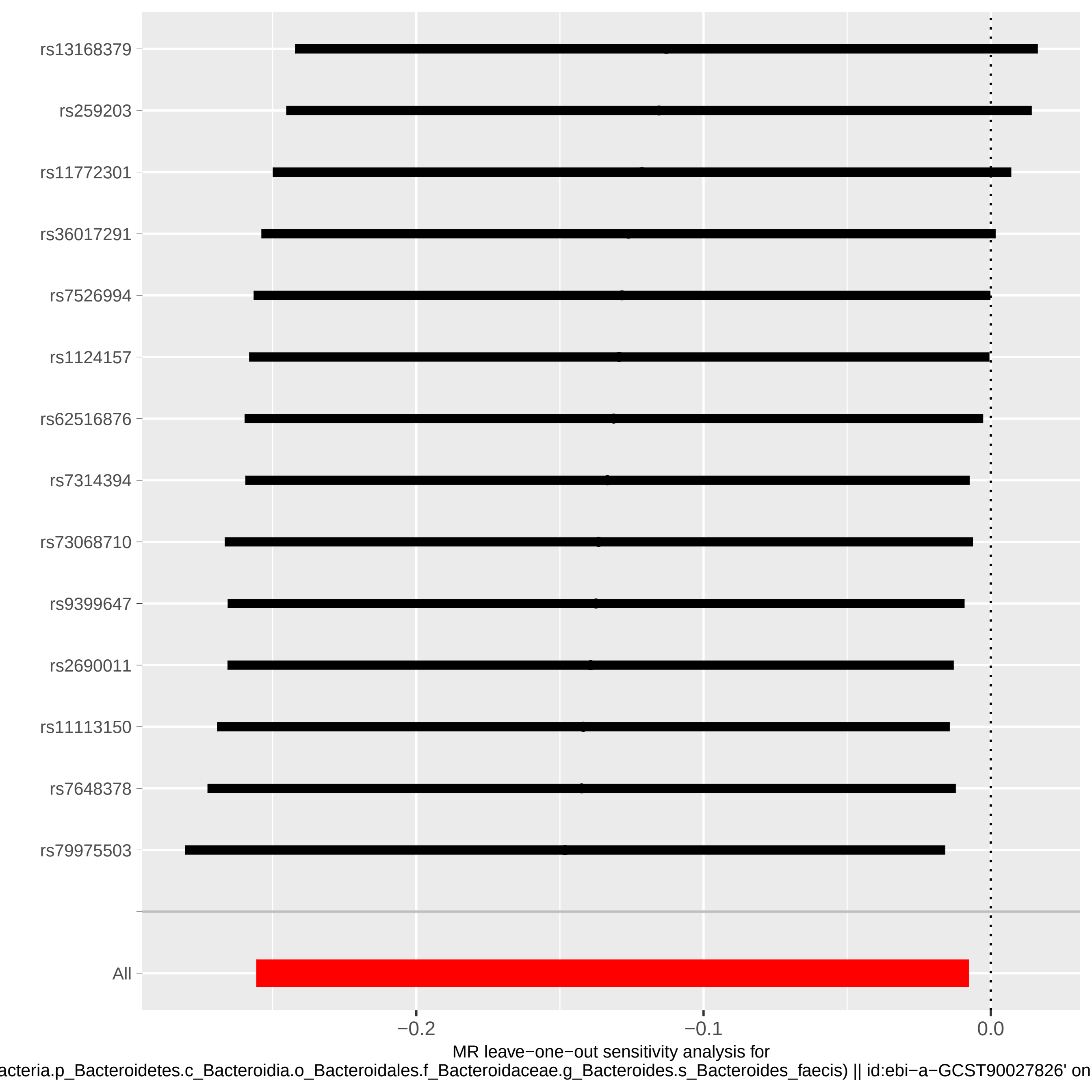

rs13168379
rs259203
rs11772301
rs36017291
rs7526994
rs1124157
rs62516876
rs7314394
rs73068710
rs9399647
rs2690011
rs11113150
rs7648378
rs79975503
All
−0.2
−0.1
0.0
MR leave−one−out sensitivity analysis for
acteria.p_Bacteroidetes.c_Bacteroidia.o_Bacteroidales.f_Bacteroidaceae.g_Bacteroides.s_Bacteroides_faecis) || id:ebi−a−GCST90027826' on
